# Acute Stress Influences Neural Circuits of Reward Processing

**DOI:** 10.3389/fnins.2012.00157

**Published:** 2012-11-01

**Authors:** Anthony J. Porcelli, Andrea H. Lewis, Mauricio R. Delgado

**Affiliations:** ^1^Department of Psychology, Marquette UniversityMilwaukee, WI, USA; ^2^Department of Psychology, Rutgers UniversityNewark, NJ, USA

**Keywords:** acute stress, cold pressor, reward processing, dorsal striatum, orbitofrontal cortex, fMRI, cortisol

## Abstract

People often make decisions under aversive conditions such as acute stress. Yet, less is known about the process in which acute stress can influence decision-making. A growing body of research has established that reward-related information associated with the outcomes of decisions exerts a powerful influence over the choices people make and that an extensive network of brain regions, prominently featuring the striatum, is involved in the processing of this reward-related information. Thus, an important step in research on the nature of acute stress’ influence over decision-making is to examine how it may modulate responses to rewards and punishments within reward processing neural circuitry. In the current experiment, we employed a simple reward processing paradigm – where participants received monetary rewards and punishments – known to evoke robust striatal responses. Immediately prior to performing each of two task runs, participants were exposed to acute stress (i.e., cold pressor) or a no stress control procedure in a between-subjects fashion. No stress group participants exhibited a pattern of activity within the dorsal striatum and orbitofrontal cortex (OFC) consistent with past research on outcome processing – specifically, differential responses for monetary rewards over punishments. In contrast, acute stress group participants’ dorsal striatum and OFC demonstrated decreased sensitivity to monetary outcomes and a lack of differential activity. These findings provide insight into how neural circuits may process rewards and punishments associated with simple decisions under acutely stressful conditions.

## Introduction

Human decision-making often occurs under stressful conditions. The type of stress exposure may be intrinsic or inherent to the decision itself (e.g., choosing between two desirable, but costly options with important consequences) or extrinsic, a pre-existing state which influences decision-making (e.g., stress exposure leading a person to use drugs as a coping mechanism). Thus, understanding how stress exposure influences decision-making is a topic of great interest. Recent efforts suggest that acute stress can modulate risk-taking in decision-making (Preston et al., 2007; Mather et al., 2009; Porcelli and Delgado, 2009), conditioning (for review, see Shors, 2004), and reinforcement learning critical to guiding future decisions (Cavanagh et al., 2010; Petzold et al., 2010). However, less is known about the impact of stress exposure on the processing of affective outcomes, a critical aspect of decision-making. The goal of the current experiment was to examine the influence of exposure to acute stress on reward-related responses in neural circuitry during the delivery of monetary rewards and punishments.

A rich animal literature has delineated a network of regions involved in processing reward-related information, also used to inform decision-making in the human brain (for review, see Schultz, 2006; Balleine et al., 2007; Haber and Knutson, 2010). This reward-related corticostriatal circuitry consists of prefrontal cortex (PFC) regions such as medial PFC and orbitofrontal cortex (OFC) as well as subcortical limbic regions involved in motivation and affect, including the dorsal and ventral striatum. The multifaceted striatum is of particular importance in coding for the subjective value of reward-related information critical to evaluation of outcomes associated with decisions (for review, see O’Doherty et al., 2004; Delgado, 2007; Rangel et al., 2008). Notably, components of the same reward-related neural circuitry have been implicated as a target of the physiological and neurochemical changes associated with engagement of the stress response.

Two complementary biological systems activated by acute stress exposure may influence brain regions involved in reward processing: the sympatho-adrenomedullary axis (i.e., the sympathetic branch of the autonomic nervous system or ANS) and the hypothalamic-pituitary-adrenal axis (HPA; for review, see Ulrich-Lai and Herman, 2009). In response to stress-related homeostatic disruption, the sympathetic ANS quickly responds with the release of catecholamines (CA; e.g., noradrenaline) from the adrenal medulla and ascending CA neurons in communication with the brainstem. As CA release in the peripheral nervous system promotes rapid excitatory changes within the body that enable an organism to deal with the source of the disruption (i.e., the classic “fight-or-flight” response; Cannon, 1915), signals of homeostatic disruption from the brainstem contribute to activation of the HPA via projections to the paraventricular nucleus of the hypothalamus. Proceeding at a slower pace, HPA activation ultimately results in the release of glucocorticoids from the adrenal cortex (i.e., cortisol in humans, corticosterone in rodents; Lupien et al., 2007).

Overall, the influence of acute stress has been studied in the context of memory and other cognitive processes (Joels et al., 2006), but less is known about the impact of stress on processing of reward-related information. One prominent idea is that stress may promote a shift from goal-oriented decision-making toward habit-based decisions that are insensitive to one’s current environment, and can be maladaptive in some contexts (Schwabe and Wolf, 2011; Schwabe et al., 2012). This is supported by studies highlighting changes in structure and function of striatal regions involved in reward-related learning and habit-based decisions (e.g., Delgado, 2007; Tricomi et al., 2009; Balleine and O’Doherty, 2010). For example, rats exposed to chronic stress exhibit marked degradation of dorsomedial striatum and medial PFC with concurrent augmentation of the dorsolateral striatum associated with sustained habitual responses to stimuli even when altered decision outcomes devalue those responses (Dias-Ferreira et al., 2009). In humans, stress-related reductions in reward-related medial PFC responses have been observed in a task involving monetary rewards or neutral outcomes (Ossewaarde et al., 2011), while exposure to acute stress has been linked to reductions in dorsomedial striatal responses to a primary reward (i.e., food; Born et al., 2009).

The current literature suggests that acute stress may modulate neural systems involved in reward processing, particularly the striatum, but a direct test of this hypothesis in humans has not yet been made. The goal of the current study was to utilize a simple reward processing paradigm known to evoke robust striatal responses to examine the influence of exposure to acute stress on outcome evaluation. A potent secondary reinforcer was used: monetary rewards and punishments. A variant of a card guessing task was employed which involved asking participants to make a choice regarding a hidden number on a virtual “card” (Delgado et al., 2000). When participants guessed correctly, they received a monetary reward. When they guessed incorrectly, they received a monetary punishment. Furthermore, rewards and punishments varied in magnitude (high or low). In past research, performance on this task has been shown to evoke robust fMRI blood-oxygen-level-dependent (BOLD) responses in striatal regions. We hypothesized that the previously characterized differential response between rewards and punishments in the striatum would be reduced after exposure to acute stress.

## Materials and Methods

### Participants

Thirty-four individuals participated in the study. Two participants were excluded from final data analysis, one due to an MRI equipment failure and the other resulting from a request to withdraw from participation. Thus, final data analysis was performed on 32 participants (16 females, 16 males; mean age = 23.41 years, SD years = 4.07). Participants responded to IRB-approved advertisements describing the study. The advertisements also indicated that compensation would be offered for their time at a rate of $25 per hour. All participants gave informed consent according to the guidelines of the Institutional Review Boards of the University of Medicine and Dentistry of New Jersey and Rutgers University.

### Procedure

#### Stress induction

Participants were exposed to acute stress in a between-subjects fashion using a variant on the traditional cold pressor task, which involves immersion of one’s hand into a container of ice-cold water. It is important to note that although water is not inherently incompatible with the MRI environment, if spilled it can be a threat to sensitive MRI equipment (such as the head coil). Additionally, even in the absence of damage due to a spill water can interfere with MRI signal due to its high proton density (Huettel et al., 2008). In the current experiment, we adapted the cold pressor test to fit the MRI environment. To administer cold pressor stress safely once participants were placed within the MRI, rather than prior to entry, an arm wrap was created from a combination of MRI-compatible dry gelpacs and maintained at a temperature of approximately 4°C. This “cold pressor arm wrap” was placed around the right hand and arm of participants assigned to the acute stress group for 2 min immediately prior to each of the two card guessing tasks. For participants assigned to the no stress group, a similar wrap created from towels (at room-temperature) was applied to control for tactile stimulation of the cold pressor arm wrap prior to each card guessing task. Hereafter, when making reference to the two groups collectively the term “experimental groups” will be used.

#### Card guessing task

**Figure 1:**
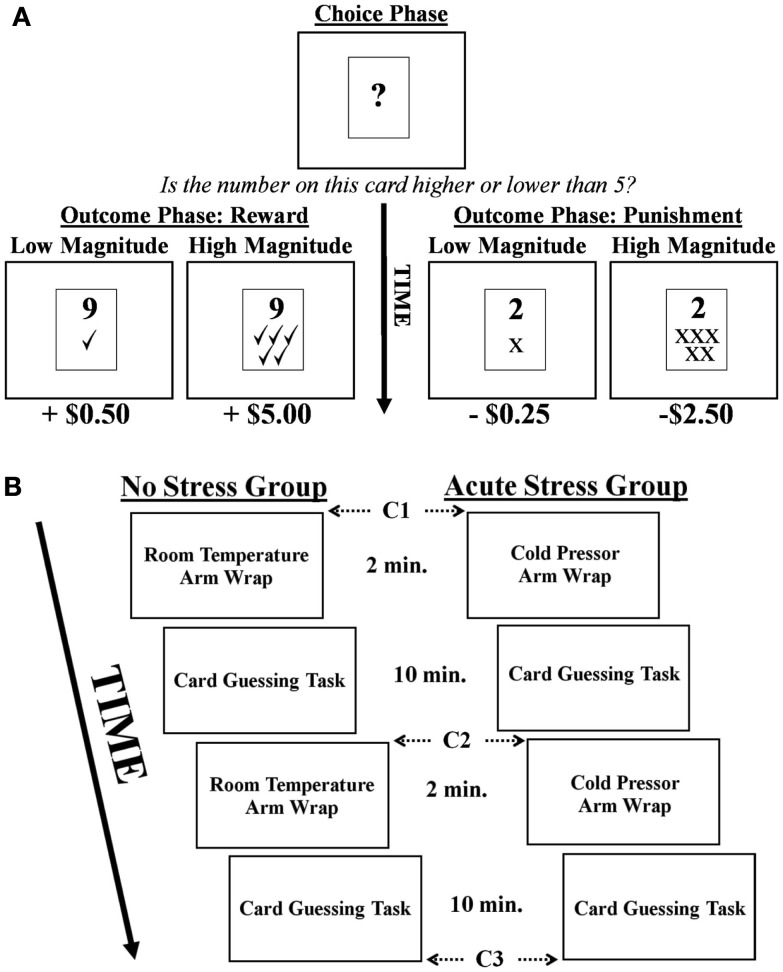
**(A)** Depiction of the card guessing task. Note, in the example above a correct choice would be “higher than five.” **(B)** Experimental timeline and cortisol sampling schedule (C = cortisol sample).

In the card guessing task (adapted from Delgado et al., 2000; Delgado et al., 2003) participants were presented with a virtual “card” upon which a question mark was printed for 2 s, representing a number between 1 and 9 (Figure 1A). Their task was to make a button press during those 2 s indicating whether they believed the number on the card was higher or lower than the number 5 (choice phase). After making their response during the 2 s choice phase, the actual number appeared on the card for 2 s (outcome phase). If participants had made a correct guess, they received a monetary reward. If their guess was incorrect, they received a monetary punishment. Rewards and punishments could be of high or low magnitude (reward: +$5.00 or +$0.50; punishment: −$2.50 or −$0.25). Importantly, values were manipulated to account for increased sensitivity to monetary losses over gains (i.e., loss aversion), thus ensuring that variations in BOLD signal related to rewards were comparable to those associated with punishments (Tversky and Kahneman, 2004). The magnitude of a reward was concurrently presented during the 2 s outcome phase via presentation of five green check marks (high magnitude) or one green check mark (low magnitude) below the card’s indicated number. Similarly, the magnitude of monetary punishments was represented by five red “×” marks (high magnitude) or one red “×” mark (low magnitude). Participants were explicitly informed as to the monetary value associated with each stimulus prior to beginning the task, but actual dollar amounts were not presented during the task (only the check and × marks). A jittered inter-trial-interval followed the outcome phase during which participants viewed a fixation lasting between 10 and 12 s, followed by the next trial.

Participants engaged in two runs of the card guessing task and were informed that they would receive compensation consistent with their performance (i.e., the outcomes they were presented with) during the card guessing task. Each run involved 40 trials with a total run time of 10 min. Participants were unaware that the outcome of each trial was predetermined such that a balanced presentation of rewards and punishments, as well as high and low magnitudes, was maintained. Thus, of the 40 trials per run 20 were associated with rewards and 20 with punishments, 10 of high/low magnitude for each valence. After completion of the experiment, participants were debriefed as to the actual nature of the task. They then completed a post-experimental questionnaire where they rated subjective stress levels associated with the arm wrap on a seven point Likert scale, as well as how the wrap made them feel (good or bad).

#### Salivary cortisol measurements

Participants were instructed to avoid eating, drinking (anything other than water), or smoking for 2 h prior to the beginning of the experiment to ensure that saliva samples were untainted. To acquire salivary cortisol data, participants were asked to moisten a Salimetrics Oral Swab (SOS) in their mouths for about 1 min by placing the SOS underneath their tongue. Upon completion of this procedure, the subject withdrew the SOS and the experimenter immediately placed it in an individual centrifuge tube. Three samples were acquired for each participant interspersed throughout the scanning session in approximately 15 min intervals, with the first sample taken after anatomical MRI scans were completed (prior to the first card guessing task). Samples two and three were acquired after each of the two blocks of the card guessing task. Samples were frozen in cold storage at −10°C, packed with dry ice and sent to Salimetrics Laboratory (State College, PA, USA) for duplicate biochemical assay analysis. An experimental timeline and cortisol sampling schedule is presented in Figure 1B. Importantly, female participants were screened for use of oral contraceptives (OC) that might influence cortisol levels (though this information was not used as an exclusionary criterion *per se*). Although 5 of the 16 female participants did report use of OC, no significant differences in cortisol levels were observed between OC and non-OC participants as measured by repeated-measures ANOVA. Furthermore, when those five participants were excluded from the imaging analysis the significance and directionality of all reported effects remained unchanged.

### fMRI acquisition and analysis

Imaging was performed on a 3T Siemens Allegra scanner equipped with a fast gradient system for echoplanar imaging. A standard radiofrequency head coil with foam padding was used to restrict participants’ head motion while minimizing discomfort. High-resolution axial images (T1-weighted MPRAGE: 256 × 256 matrix, FOV = 256 mm, 176 1 mm axial slices) were obtained from all subjects. Functional images (single-shot gradient echo EPI sequence; TR = 2000 ms; TE = 25 ms; FOV = 192 cm; flip angle = 80°; matrix = 64 × 64; slice thickness = 3 mm) were acquired during performance on the two card guessing task runs. Data were then preprocessed and analyzed using BrainVoyager QX software (version 2.2, Brain Innovation, Maastricht, Netherlands). Preprocessing involved motion correction (six-parameter, three-dimensional), spatial smoothing (4-mm FWHM), voxel-wise linear detrending, high-pass filtering of frequencies (three cycles per time course) and normalization to Talairach stereotaxic space (Talairach and Tournoux, 1988).

General linear models (GLM) were defined at the single-subject level in which predictors were regressed onto the dependent variable of BOLD changes within the brain. Two separate models were generated. In model 1 (outcome valence only), two predictors modeled the outcome phase of the card guessing task based on whether participants had received a rewarding outcome (gain of money) or punishing outcome (loss of money) after their choice. For model 2 (outcome valence and outcome magnitude), the magnitude of rewards and punishments were included, resulting in a model comprised of four predictors: high magnitude reward, low magnitude reward, high magnitude punishment, and low magnitude punishment. In both models, motion parameters generated during fMRI data preprocessing were included as covariates of no-interest (to control for head motion), as was a missed-trial predictor. Two second-level random effects GLMs were then performed.

Based on the random effects GLMs whole-brain statistical parametric maps were generated. Given *a priori* patterns of BOLD signal defined by a similar contrasts in past work (for review, see Delgado, 2007) it was thought that a Reward – Punishment contrast would best highlight task-related alterations in BOLD signal in regions of the brain known to be involved in processing reward-related information. Using model 1 (outcome valence only) a whole-brain two-tailed contrast was performed on outcome phase BOLD in which rewards and punishments were received (Reward – Punishment), and the difference in BOLD associated with this contrast was contrasted along the between-subjects factor of experimental group (No Stress vs. Acute Stress). Thus, this analysis highlighted brain regions responsive to outcome valence that significantly differed between experimental groups. In a similar whole-brain analysis using model 2, a contrast of high and low magnitude outcomes across outcome valence was performed ([High Reward + High Punishment] – [Low Reward + Low Punishment]) and the difference in BOLD associated with this contrast was computed along the between-subjects factor of experimental group (No Stress vs. Acute Stress). Therefore, this analysis examined brain regions responsive to the magnitude of monetary outcomes that significantly differed between experimental groups.

The resultant contrast maps were then examined to identify statistically significant clusters of activation at a threshold of *p* < 0.005, with a contiguity threshold of 5^3^ mm voxels. Correction for multiple comparisons was verified through the use of cluster-size thresholding (Forman et al., 1995; Goebel et al., 2006). Thus, only clusters of a sufficient extent so as to be associated with a cluster-level false-positive rate of α = 0.05 remained in the analysis. Additionally, an exploratory analysis of the possible role of participants’ sex was performed in *a priori* regions of interest given previous sex-related effects observed in the literature (e.g., Lighthall et al., 2011). Specifically, parameter estimates were extracted from significant clusters resultant from both contrasts and examined for potential interactions with sex. Importantly, all *post hoc* tests within each family of analyses were corrected for multiple comparisons via sequential Bonferroni correction (Holm, 1979).

## Results

### Reaction time data

A two-tailed independent *t*-test was performed to examine differences in reaction time in the card guessing task between experimental groups. No significant difference was observed in reaction times for the acute stress (*M* = 623.31, SEM = 45.91) vs. no stress (*M* = 633.77, SEM = 43.81) groups, *t*(30) = 0.17, *p* > 0.15, *d* = 0.06.

### Subjective stress ratings

Post-experimental subjective ratings of perceived stress experience were examined between acute stress and no stress experimental groups via independent *t*-tests. These included ratings of how the cold pressor arm wrap made participants feel (good to bad) and how stressful (high to low) the experience was. Compared to the no stress group, the acute stress group rated the arm wrap as feeling significantly worse [*t*(30) = 4.42, *p* < 0.001, *d* = 1.56] and more stressful [*t*(30) = 3.46, *p* < 0.01 *d* = 1.22].

### Salivary cortisol data

**Figure 2:**
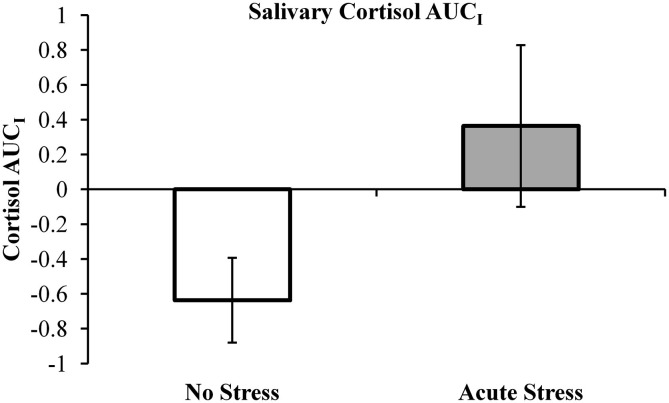
**Salivary cortisol area under the curve with respect to increase (AUC_I_) by experimental group**. Note, negative AUC_I_ values (indicating a decrease in salivary cortisol over the course of the experiment) were retained as an “index of decrease” as recommended by Pruessner et al. (2003).

Salivary cortisol data were excluded for three participants, in one case due to a corruption of the samples and in two cases due to an inability to acquire samples during MRI scanning. Thus, cortisol analyses were conducted on 29 of the 33 participants (13 no stress, 16 acute stress). Mean salivary cortisol levels (in nmol/L) for all three samples by experimental group are reported in Table 1. A 3 (Sample 1, 2, or 3) × 2 (Experimental Group: No Stress vs. Stress) repeated-measures ANOVA was performed, but no significant interaction between sample and experimental group was observed, *F*(2, 54) = 1.77, *p* = 0.18, ${\text{η}}_{p}^{2}=$ 0.061. Area under the curve with respect to increase (AUC_I_) was calculated using the trapezoidal method for both experimental groups. This measure is useful in that it represents both time-related changes in salivary cortisol levels as well as the overall intensity of said changes (Pruessner et al., 2003). A one-tailed independent *t*-test between AUC_I_ for the experimental groups (No stress vs. Acute Stress) indicated a significant increase in cortisol levels for those participants who were exposed to acute stress, *t*(27) = 1.78, *p* < 0.05, *d* = 0.69 (Figure 2). No significant correlations were observed between cortisol and imaging data presented below.

**Table 1:** **Mean salivary cortisol levels in nmol/L at baseline, after task run 1, and after task run 2 by experimental group (Mean ± SEM)**.

Sample (nmol/L)	Experimental group	
	No stress	Acute stress
Baseline (min)	3.93 ± 0.52	3.80 ± 0.28
Post-baseline 1 (∼15)	3.61 ± 0.45	4.23 ± 0.54
Post-baseline 2 (∼30)	3.31 ± 0.38	3.67 ± 0.42

### fMRI results

#### Outcome valence: reward – punishment by experimental group contrast

In the no stress group, multiple brain regions demonstrated greater BOLD signal associated with the reward – punishment contrast than were observed in the acute stress group (see Table 2). Prominently featuring among these regions were the dorsal striatum (specifically the right caudate nucleus and left putamen) and the left OFC.

**Table 2:** **Brain regions that demonstrated differences by experimental group (No Stress vs. Acute Stress) for Reward – Punishment and High – Low Magnitude contrasts (*p* < 0.005, corrected)**.

Activated region	Laterality	Talairach coordinates			Voxel count (mm^3^)	*T*-value
		*x*	*y*	*z*	
**REWARD – PUNISHMENT (NO STRESS > ACUTE STRESS GROUP)**						
Superior parietal lobule (BA 7)	R	38	−65	48	355	4.24
Middle frontal gyrus (BA 6)	R	41	13	45	239	4.74
Inferior parietal lobule (BA 40)	R	41	−38	42	2693	5.58
Middle frontal gyrus (BA 9)	R	35	31	30	1382	5.48
Middle frontal gyrus (BA 9)	L	−28	13	30	135	4.70
Precentral Gyrus (BA 6)	R	35	4	27	152	5.25
Caudate (dorsal striatum)	R	14	4	18	206	3.74
Putamen (dorsal striatum)	L	−22	4	6	138	4.43
Orbitofrontal cortex (BA 47)	L	−40	43	−6	170	3.81
Middle temporal gyrus (BA 21)	R	53	−32	−9	188	4.35
Inferior temporal gyrus (BA 37)	R	53	−53	−12	137	4.13
Inferior temporal gyrus (BA 20)	L	−55	−26	−18	146	4.31
Fusiform gyrus (BA 20)	L	−58	−14	−24	186	4.22
**REWARD – PUNISHMENT (ACUTE STRESS > NO STRESS GROUP)**						
Cuneus/posterior cingulate (BA 18/31)	L	−25	−56	6	177	−4.22
**HIGH – LOW MAGNITUDE (NO STRESS > ACUTE STRESS GROUP)**						
Inferior frontal gyrus (BA 45)	L	−58	13	18	873	5.77

*BA, Brodmann Area; L, left; R, right*.

**Figure 3:**
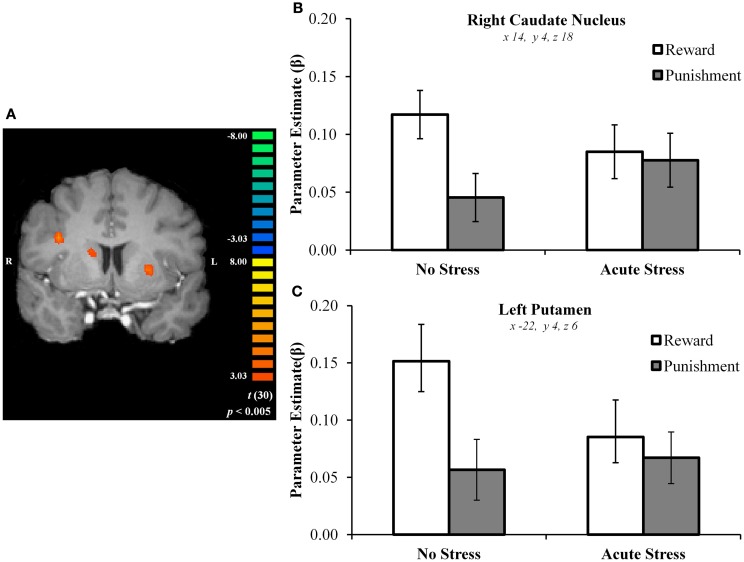
**(A)** Right caudate and left putamen clusters exhibiting greater BOLD signal for no stress over acute stress participants. **(B)** Right caudate nucleus outcome valence × experimental group parameter estimates. **(C)** Left putamen outcome valence × experimental group parameter estimates.

**Figure 4:**
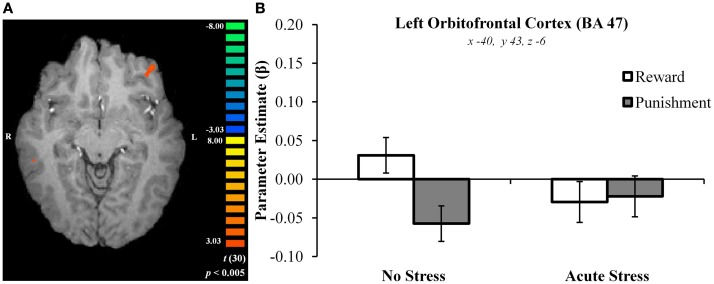
**(A)** Left orbitofrontal cortex cluster exhibiting greater BOLD signal for no stress over acute stress participants. **(B)** Left orbitofrontal cortex outcome valence × experimental group parameter estimates.

In the right caudate, *post hoc* paired *t*-tests suggested that BOLD signal in the no stress group was significantly greater for rewards than punishments, *t*(15) = 5.69, *p* < 0.001, *d* = 0.88 (Figures 3A–C). No significant difference was observed in the acute stress group, *t*(15) = 0.74, *p* > 0.15, *d* = 0.08. A similar pattern of BOLD signal was observed in the left putamen [no stress, *t*(15) = 6.57, *p* < 0.001, *d* = 0.73; acute stress, *t*(15) = 1.24, *p* > 0.15, *d* = 0.18] and left OFC [no stress, *t*(15) = 6.80, *p* < 0.001, *d* = 1.15; acute stress, *t*(15) = 0.37, *p* > 0.15, *d* = 0.06; see Figure 4]. Thus, whereas the no stress group demonstrated a clear response to rewards over punishments in these regions, the group that had been exposed to acute stress exhibited a lack of responsiveness to reward-related information. All significant *t*-tests survived sequential Bonferroni correction.

Parameter estimates for these three regions in the acute stress group were then examined in a second analysis for the presence of magnitude-related effects (an orthogonal factor not included in the original contrast) in reward and punishment trials. In the right caudate, *post hoc* paired *t*-tests suggested that BOLD signal in the acute stress group was significantly greater for rewards over punishments for outcomes of high magnitude, *t*(15) = 2.79, *p* < 0.05, *d* = 0.31, but not low magnitude, *t*(15) = −1.37, *p* > 0.15, *d* = −0.25. A similar pattern was observed within the left putamen. Acute stress group BOLD differentiated between high magnitude outcomes, *t*(15) = 2.84, *p* < 0.05, *d* = 0.43, but not low magnitude outcomes, *t*(15) = −0.83, *p* > 0.15, *d* = −0.20. Notably, in contrast to the above regions the left OFC in the acute stress group did not significantly differentiate between outcomes of either magnitude [high: *t*(15) = 1.25, *p* > 0.15, *d* = 0.27; low: *t*(15) = −1.71, *p* > 0.10, *d* = −0.34]. All significant *t*-tests survived sequential Bonferroni correction.

To examine whether or not a difference was present in the stress effect between the two task runs, a region of interest (ROI) analysis was performed investigating right dorsal striatum, left putamen, and left OFC BOLD signal between runs 1 and 2 (using ROIs from the original whole-brain analysis). Parameter estimates extracted from the three aforementioned ROIs were examined via 2 (Run: Run 1 vs. Run 2) × 2 (Outcome Valence: Reward vs. Punishment) × 2 (Experimental Group: No Stress vs. Acute Stress) repeated-measures ANOVA for the purpose of establishing whether or not a difference in BOLD existed as a function of run. No significant interaction was observed between run, experimental group, and outcome valence in the right dorsal striatum, *F*(1, 30) = 0.001, *p* > 0.15, ${\text{η}}_{p}^{2}=$ 0.000, left putamen, *F*(1, 30) = 0.77, *p* > 0.15, ${\text{η}}_{p}^{2}=$ 0.025, or left OFC, *F*(1, 30) = 0.31, *p* > 0.15, ${\text{η}}_{p}^{2}=$ 0.010, suggesting that the previously discussed effects were not different between runs.

#### Outcome magnitude: high – low by experimental group contrasts

A single brain region was associated with increased BOLD signal for no stress participants in the outcome magnitude contrast: the left inferior frontal gyrus (BA45). *Post hoc* paired *t*-tests indicated that no stress participants showed greater BOLD responses to high over low magnitude outcomes (across outcome valence), *t*(15) = 4.77, *p* < 0.001, *d* = 0.76. Acute stress participants, however, demonstrated a trend (which did not survive Bonferroni–Holm correction) toward the reverse pattern – increased BOLD for low over high magnitude outcomes, *t*(15) = −1.98, *p* < 0.10, *d* = −0.38.

#### Exploratory analyses: sex effects

Salivary cortisol AUC_I_ was examined via univariate ANOVA for sex-related differences in cortisol increases by experimental group. No significant main effect of sex on salivary cortisol was observed, *F*(1, 25) = 0.52, *p* = 0.48, ${\text{η}}_{p}^{2}=$ 0.020, nor was a significant sex by experimental group interaction observed, *F*(1, 25) = 0.03, *p* = 0.87, ${\text{η}}_{p}^{2}=$ 0.001. Parameter estimates extracted from significant clusters in both contrasts were subjected to a series of 2 (Outcome valence: Reward vs. Punishment) × 2 (Experimental Group: No Stress vs. Acute Stress) × 2 (Sex: male vs. female) repeated-measures ANOVAs to explore -the possible role of sex in stress-related differences in processing of reward-related information. In the right caudate a trend towards a significant experimental group × sex interaction was observed, *F*(1, 28) = 3.27, *p* < 0.10, ${\text{η}}_{p}^{2}=$ 0.105. *Post hoc* independent *t*-tests indicate that no stress group female participants exhibited greater BOLD signal overall for all outcomes than did males, *t*(14) = −2.57, *p* < 0.05, *d* = −1.28. In contrast acute stress group males’ BOLD was elevated as compared to the no stress group whereas females’ was reduced, resulting in a non-significant difference between the sexes, *t*(14) = 0.44, *p* > 0.15, *d* = 0.22. No other brain regions exhibited trending or significant sex effects.

## Discussion

In this study, we sought to investigate how exposure to acute stress influenced neural responses to monetary rewards and punishments. We used a between-subjects approach and tested performance of participants after application of a cold pressor procedure (acute stress group), compared to a control procedure (no stress group) during two runs of a simple card guessing paradigm previously found to yield robust striatal activation to reward responses (e.g., Delgado et al., 2000). Salivary cortisol data and subjective stress ratings confirmed that the stressor (i.e., cold pressor arm wrap adapted for fMRI) was effective. Participants exposed to acute stress exhibited a marked alteration in neural responses to monetary rewards and punishments. Whereas dorsal striatal BOLD signal within the right caudate nucleus and left putamen differentiated between rewarding and punishing outcomes in no stress participants, this was not the case in acute stress participants. A similar pattern of activity was observed in the left OFC. Notably, high magnitude rewards and punishments were resilient to the stress effect in striatal regions but not within OFC. Taken together, these results suggest that exposure to acute stress affects reward-related processing in the dorsal striatum and OFC.

This study complements and augments a growing literature examining the influence of acute stress on human decision-making by attempting to characterize striatal responses to outcome processing under stress. Previous studies have shown modulation of striatal response under stress using different paradigms and reinforcers. For instance, acute stress-related reductions in putamen responses to primary rewards (food images) have been observed (Born et al., 2009), which complements the outcome processing of secondary reinforcers in the current paradigm observed in both caudate and putamen. The consequences of decreased sensitivity to reward processing is a question for future research, but it is informed by a recent study suggesting that increased life stress and reduced ventral striatum reactivity to rewards (i.e., positive performance feedback) interact to predict low levels of positive affect on a depression scale (Nikolova et al., 2012). This converges with previous behavioral work indicating a reduction in responsiveness to rewards under acute stress (Bogdan and Pizzagalli, 2006) which the current study builds upon with the observation of reductions in reward-related responses in the dorsal striatum after acute stress exposure.

An interesting observation from our study is that the stress modulation effect was observed in the dorsal, but not the ventral, striatum. A null finding, however, should not be interpreted as a lack of stress modulation of ventral striatum responses (in fact, stress-related ventral striatal activation has been observed in a non-reward-related task; Pruessner et al., 2008); rather, it highlights the sensitivity of dorsal striatum activity to stress modulation (e.g., Sinha et al., 2005). The dorsal striatum, particularly the caudate, has often been found to be robustly recruited by the reward paradigm used in the current paper (for review, see Delgado, 2007). Further, the dorsal striatum has been posited to function as an “actor” that maintains information about action-contingent response-reward associations to guide future decisions based on the outcomes of past ones, while the ventral portion a “critic” that predicts possible future rewards (O’Doherty et al., 2004; Tricomi et al., 2004). Thus, by impairing the ability of the dorsal striatum to distinguish between rewarding vs. punishing outcomes, acute stress may interfere with the use of information provided by past decisions to guide future choices.

Within the dorsal striatum itself, a functional subdivision suggests that the medial portion of the dorsal striatum is involved in flexible, goal-oriented, and action-contingent decision-making whereas the lateral portion mediates habitual and stimulus bound decisions (Balleine et al., 2007; Tricomi et al., 2009). In the current experiment, it is plausible that stress-related changes in BOLD signal observed in the dorsomedial striatum (i.e., caudate) and dorsolateral striatum (i.e., putamen) mark the beginning of a shift from goal-directed to habitual processing of decision outcomes, although further work is necessary to test this hypothesis using an affective learning paradigm. The hypothesis is consistent with previous behavioral work in support of stress’ ability to shift decision-related processing from goal-oriented to habitual (i.e., as in instrumental conditioning; Schwabe and Wolf, 2011). Importantly, decreased sensitivity to reward processing in the dorsal striatum may have important clinical applications with respect to decision-making and one’s general affect. For instance, stress- and drug-cue associated alterations in dorsal striatal function have been implicated in relapse in drug/alcohol addiction (Sinha and Li, 2007) and reduced dorsal striatal responses to rewards have been observed in unmedicated individuals suffering from major depressive disorder (Pizzagalli et al., 2009).

Another brain region implicated in processing of reward-related information is the OFC, which in this experiment also exhibited alterations in responsiveness to rewards and punishments. It has been suggested that this region may be involved in outcome evaluation by coding for the subjective value of said decision outcomes (O’Doherty et al., 2001a). For example, increases in OFC BOLD have been observed during delivery of pleasant as compared to aversive gustatory stimuli (O’Doherty et al., 2001b). Although stress-related reductions in brain function during reward processing have been somewhat studied in neighboring prefrontal regions such as the medial PFC (Ossewaarde et al., 2011) OFC has received less attention in this regard, making it an ideal topic for future research. This is especially the case with respect to the effects of stress on drug addiction, as this region may play a role in the inability of addicts to alter their behavior based on likely outcomes or consequences – leading to relapse (Schoenbaum and Shaham, 2008). A notable exception is a recent study suggesting the necessity of concurrent CA and glucocorticoid activation in reductions in OFC sensitivity to reward-related information (e.g., Schwabe et al., 2012).

With respect to the mechanism underlying the findings of the current study, several plausible interpretations can be considered. It has been established that glucocorticoid responses to cold pressor stress are less extreme than have been observed in other stress induction techniques, such as stressors involving a psychosocial component (e.g., McRae et al., 2006; Schwabe et al., 2008). In the current study, this is reflected by mild-to-moderate acute stress group increases in cortisol. In contrast, it is likely that sympathetic ANS activation remains comparable between cold pressor and other forms of stress. Another consideration is that in the current study initial acute stress exposure occurred immediately prior to the first card guessing task, followed 15 min later by a second stress exposure and card guessing task. As the effects of glucocorticoid release in this type of paradigm would likely be genomic (i.e., slow and long-lasting; Sapolsky et al., 2000) it is possible that they did not influence brain function in the first task run. Yet, the observed decrease in striatal and OFC responsiveness to reward-related information was present in both task runs. Further, as stress-related increases in cortisol were modest here it is possible that glucocorticoids did not contribute to the effect at all. Thus, lack of data that can speak to the dynamics of sympathetic ANS activation (e.g., skin conductance or salivary alpha amylase; Rohleder et al., 2004) constitutes a study limitation. While the paradigm employed here was not designed to address these issues, it is likely that contextual factors including the nature and timing of stress exposure and the mode of reward-related information involved in the task play an important role.

Some studies suggest that sex differences may play a role in stress-related alterations in striatal reward processing. For example, studies examining the influence of acute stress on risk-tasking have established fluctuations in dorsal striatal function as a function of gender (Lighthall et al., 2009, 2011). There participants performed the Balloon Analog Risk Task, which involves making a button press to expand a virtual balloon for monetary rewards. With each button press, more money is gained – but at a certain point the balloon will explode. Thus, participants risk losing all winnings if they continue to expand the balloon to gain additional rewards. It was observed that under acute stress males take more risks and exhibit increases in dorsal striatal function, whereas females show the reverse pattern, as compared to no stress participants. In the current study, a trend toward a sex difference along similar lines was also observed in the dorsal striatum – though to a lesser degree. No stress females’ BOLD for outcomes was elevated above males’. While BOLD signals to outcomes did decrease for acutely stressed females and increased for males, the result was more extreme in the Lighthall et al. (2009, 2011) studies. This may relate to the fact that risk-taking tasks such as the balloon task involve anticipation of potential outcomes in addition to an outcome evaluation component, while also requiring participants make complex choices balancing potential rewards against potential punishments. It may be the case that the simple outcome evaluation paradigm used in our study is less sensitive to sex differences than more dynamic and complex risk-taking paradigms.

In sum, this paper used a novel approach to induce stress in the fMRI scanner (the cold pressor arm wrap) and observed that exposure to acute stress modulated reward-related circuitry. Specifically, participants under stress showed decreased differential responses to reward and punishment in the dorsal striatum and OFC. Future studies may try to probe if this decreased differential response is driven by a diminished response to rewards (as previously observed in the literature, e.g., Born et al., 2009) or an increase in sensitivity to negative outcomes. Further, additional research is needed to clarify how neural responses to these distinct reinforcers might influence subsequent decision-making under stress.

## Conflict of Interest Statement

The authors declare that the research was conducted in the absence of any commercial or financial relationships that could be construed as a potential conflict of interest.
